# Community empowerment and mental wellbeing: longitudinal findings from a survey of people actively involved in the big local place-based initiative in England

**DOI:** 10.1093/pubmed/fdac073

**Published:** 2022-07-30

**Authors:** N Akhter, V J McGowan, E Halliday, J Popay, A Kasim, C Bambra

**Affiliations:** Fuse – UKCRC Centre for Translational Research in Public Health, Newcastle Upon Tyne, NE1 7RU, UK; Department of Anthropology, Durham University, Durham DH1 3LE, UK; Fuse – UKCRC Centre for Translational Research in Public Health, Newcastle Upon Tyne, NE1 7RU, UK; Population Health Sciences Institute, Newcastle University, Newcastle-upon-Tyne, NE1 4LP, UK; Division of Health Research, Faculty of Health & Medicine, Lancaster University, Bailrigg, Lancaster LA1 4YG, UK; Division of Health Research, Faculty of Health & Medicine, Lancaster University, Bailrigg, Lancaster LA1 4YG, UK; Fuse – UKCRC Centre for Translational Research in Public Health, Newcastle Upon Tyne, NE1 7RU, UK; Department of Anthropology, Durham University, Durham DH1 3LE, UK; Fuse – UKCRC Centre for Translational Research in Public Health, Newcastle Upon Tyne, NE1 7RU, UK; Population Health Sciences Institute, Newcastle University, Newcastle-upon-Tyne, NE1 4LP, UK

**Keywords:** community, control, deprivation, health inequality, social determinants, socioeconomic status

## Abstract

**Background:**

Community empowerment initiatives are receiving increased interest as ways of improving health and reducing health inequalities.

**Purpose:**

Longitudinally examine associations between collective control, social-cohesion and mental wellbeing amongst participants in the Big Local community empowerment initiative across 150 disadvantaged areas of England.

**Methods:**

As part of the independent Communities in Control study, we analysed nested cohort survey data on mental wellbeing (Short Warwick Edinburgh Mental Wellbeing Scale—SWEMWBS) and perceptions of collective control and social-cohesion. Data were obtained in 2016, 2018 and 2020 for 217 residents involved in the 150 Big Local areas in England. Adjusted linear mixed effect models were utilized to examine changes in SWEMWBS over the three waves. Subgroup analysis by gender and educational level was conducted.

**Results:**

There was a significant 1.46 (0.14, 2.77) unit increase in mental wellbeing score at wave 2 (2018) but not in wave 3 (2020) (0.06 [−1.41, 1.53]). Across all waves, collective control was associated with a significantly higher mental wellbeing score (3.36 [1.51, 5.21]) as was social cohesion (1.09 [0.19, 2.00]). Higher educated participants (1.99 [0.14, 3.84]) and men (2.41 [0.55, 4.28]) experienced significant increases in mental wellbeing in 2018, but lower educated participants and women did not.

**Conclusion:**

Collective control and social cohesion are associated with better mental wellbeing amongst residents engaged with the Big Local initiative. These health benefits were greater amongst men and participants from higher educational backgrounds. This suggests that additional care must be taken in future interventions to ensure that benefits are distributed equally.

## Background

Community empowerment initiatives have gained greater attention in recent years as potentially effective approaches to improve health and reduce inequalities in disadvantaged areas.^1^ These initiatives are built upon theoretical foundations^2^ and empirical evidence that demonstrates how control over one’s life is a significant determinant of health outcomes.^3–5^ Indeed, the social gradient in health is, in part, produced by variations in how much control people have over their lives and where they live.^6^ There is now increasing evidence demonstrating positive associations between collective control, social cohesion and improvements in health outcomes.^5^ For example, a systematic review of collective empowerment group-based micro-financing interventions found they were associated with positive health and social outcomes.^7^ However, much of this research is cross-sectional.^8^ The research reported here adds a longitudinal dimension to this evidence base.

Furthermore, there is less empirical evidence on the mechanisms by which collective control and social cohesion result in improvements in health and social outcomes. Yet, it is precisely the identification of these pathways that will produce effective actions to redress inequalities in health.^9^ Such processes and mechanisms can occur via both direct and indirect pathways.^10^ Direct pathways to health improvement are hypothesized to arise from community empowerment initiatives where residents engage in neighbourhood activities that, for example, prevent toxic waste facilities being placed in their area^10^ or maintain services threatened by state disinvestment.^11^ Indirect routes can arise if these collective activities increase social cohesion^5^ or increase a community’s sense of control over where they live.^5^^,^^10^ The latter is examined in the research reported here.

In our analysis of the 2016 baseline survey of participants in the Big Local (BL) community empowerment initiative, we demonstrated that people with higher levels of collective control and area-belonging had better mental wellbeing.^12^ In related work, we identified that active involvement with the BL led to increased feelings of belonging to the places people live, which was associated with improved mental wellbeing.^13^ Furthermore, although our findings highlighted that pathways to health improvement are complex, there were clear indications that when residents reported no improvements in collective control or social cohesion, there was a decline or no improvement in mental wellbeing.^13^

This paper presents the findings from analysis of a longitudinal nested cohort survey of people actively involved in BL conducted over 4 years and three waves (2016, 2018, 2020), examining in further detail the mechanisms of collective control, social cohesion and mental wellbeing arising from the BL initiative. This research was part of a larger independent Communities in Control (CiC) study (https://communitiesincontrol.uk/) that uses a mixed methods approach to evaluate the health and social impacts of BL.

## Methods

### The BL initiative

BL is a place-based resident-led initiative funded by the National Lottery Community Fund (formerly Big Lottery Fund) and managed by a charitable organization, Local Trust (LT). The BL initiative, which runs from 2010 to 2026, provides 150 disadvantaged areas in England with at least £1 million to support residents in making their neighbourhood a better place to live. There is considerable flexibility in how residents decide to use the funds but a core element of the initiative is placing residents in control over these decisions. This is done through the development of a BL partnership in each area, which must be comprised of at least 51% resident membership but can also include other local stakeholders (such as professionals who work in the area, people who were previously residents but have moved, relatives of residents, people who live in a neighbouring area, or people with other connections to the community). Although some members of the partnership boards do not live in the BL area they either support residents in making decisions or they are directly involved with decision-making. Moreover, the funding is accompanied by the provision of support and guidance from LT, which aims to build capabilities among residents including sharing information and developing skills (http://www.localtrust.org.uk).

### Data sources

This research used data from three waves (2016, 2018, 2020) of a biannual survey conducted by LT of all BL partnership members in all 150 BL areas across England. BL partnerships may have members from local stakeholders (e.g. the church, the NHS or third sector) but the majority must be BL residents. The Partnership Members survey is a repeat cross-sectional survey but we were able to construct a small nested cohort of linked individual records of those who participated across all three waves (*n* = 217, of which > 70% were residents). LT manage this survey biannually between May and August each year via an online and/or postal questionnaire and from 2016 onwards we were allowed to include additional questions on mental wellbeing (Short Warwick Edinburgh Mental Wellbeing), collective control and social cohesion.

### Obtaining and managing the data

LT collated the survey responses and sent them to the research team in an anonymized SPSS spreadsheet in October 2016, 2018 and 2020. They uploaded the anonymized data to a shared Box folder only accessible to named collaborators. Individual records were linked over the three waves via unique numerical identifiers for the purpose of the nested cohort. The data were stored in electronic form on secure university servers and were accessed through password-protected networked PCs and laptops.

### Variables

The survey collected data on the characteristics of BL partnership members (demographic data, socio-economic status [education]), perception of collective control and perception of the BL area, levels of participation (number of unpaid hours per week on BL activities) and self-perceived mental wellbeing using the validated measure Short Warwick Edinburgh Mental Wellbeing Scale (SWEMWBS).

The SWEMWBS (mental wellbeing) is designed to measure positive mental health states (as opposed to symptoms of mental ill-health such as anxiety and depression). Questions include the degree to which a participant ‘feels useful’, ‘feels relaxed’, ‘feels with problems well’, ‘feels close to other people’, ‘feels able to make up my mind about things’. The scale has seven domains and scores range from 7 to 35 and higher scores indicate higher positive mental wellbeing. The scale has been validated for the general population.^12^

At baseline, we examined any association with mental wellbeing with: (i) whether respondents felt able to influence decisions affecting their area, either collectively with others (collective control) or as individuals (individual control); (ii) perceptions of social-cohesion around involvement (feels got to know more people in the area, feels more connected, feels more positive about BL area, feels stronger sense of community) and (iii) area perception (feels people in the BL can be trusted; feels people in their BL are willing to help each other; feels belong to the area); and (iv) hours of involvement amongst participants.^12^ Only those explanatory variables significantly associated with mental wellbeing at baseline were then included in the follow-up analyses—collective control, social cohesion (people in area are willing to help) and hours of involvement (as time varying variables, specific for each wave).^12^ We also included resident status (lives in BL area/not).

### Population sample

In 2016, potential respondents were identified using a common sampling frame: all BL partnership members who provided contact details for the annual partnership review carried out by LT were approached via email (for an online questionnaire submission) or could be given one by the BL rep supporting their partnership. The aim was to reach as many partnership members as possible. This gave a total baseline potential sample of over 1600 partnership members across all 150 BL areas. About 862 participants submitted a completed questionnaire in 2016 (a baseline response rate of over 50%), 1011 in wave 2 (63%) and 1023 at wave 3 (64%). These repeat cross-sections provided the basis for a small nested cohort, whereby individual records were linked over the three waves; 217 participants (of which > 70% were residents) provided linked data over all three waves and comprise the nested cohort analysed in this paper.

### Analysis

We examined whether mental wellbeing changed over time across the three waves (wave 2 compared with baseline and wave 3 compared with baseline) using the nested cohort design. Our baseline analysis has previously been published in this journal.^12^ For the 2018 and 2020 data, we examined associations over time with those explanatory variables that were significant at baseline (collective control, social cohesion, hours involved, resident status). Linear mixed effect models (accounting for the clustering of participants within sites and the repeated measures per participants across the three waves) examined changes in mental wellbeing over the three waves. The different waves were treated as categorical variables. Baseline age categories, sex, highest educational qualification and ethnicity were held as constants, whereas the other variables (collective control, willingness to help each other, resident status and hours volunteered) were used as wave-specific time-varying factors. The most parsimonious model was selected using a likelihood ratio test.

We also investigated whether there were differences in effects for our primary outcome across a number of pre-defined sub-groups. Firstly, we investigated potential effects on health inequalities by analysing whether any mental wellbeing effects differed by education or gender. Secondly, we investigated any differences in terms of levels of participation in the BL (measured using hours involved) to see if there was a graded effect of participation. Thirdly, we also examined differences by resident versus non-resident status of BL partnership members. All analyses used baseline as a reference to compare the change over time for the cohort between (a) baseline and wave 2 [2018] and (b) baseline and wave 3 [2020]. The presented models examine the associations between mental wellbeing and the explanatory factors across all waves together (baseline, wave 2, wave 3) and only included wave-specific results when the associations varied between waves.

The cohort analysis performed utilized a likelihood-based mixed effects model, which assumes that data are missing at random. Missing data for SWEMWBS were 5% and 11% for the 2018 and 2020 cohort waves, respectively. Missing data rates were tabulated and examined. Only gender predicted missingness and so we adjusted for this in the main models.

## Results

Table 1 summarizes the socio-demographic, health and explanatory characteristics of the 217 nested cohort survey respondents. There were a higher proportion of women than men, and those aged 45–64 made up the majority (50.5%). Most survey respondents were White (91.5%): only 8.5% were from Black and Minority Ethnic groups and a sizeable minority (37.8%) had one or more degrees. The mean SWEMWBS score was 24. 5 (±4.5) at baseline, 25.0 (±4.0) at wave 2 and 23.9 (±4.0) at wave 3. The vast majority of participants were residents of the BL area (e.g. > 76% at wave 3) and the average hours volunteered per week were > 7.

**Table 1:** Socio-demographic, health and explanatory data for cohort participants (*n* = 217)

Variable	Categories	2016% (*n*)	2018% (*n*)	2020% (*n*)
Age	≤29 years	0.5 (1)		
	30–44 years	15.7 (34)		
	45–64 years	50.5 (109)		
	≥65 years	33.3 (72)		
	Total	100.0 (216)		
Sex	Female	56.0 (121)		
	Male	44.0 (95)		
	Total	100.0 (216)		
Ethnicity	BAME	8.5 (18)		
	White	91.5 (194)		
	Total	100.0 (212)		
Highest education	No degree	62.2 (125)		
	Degree	37.8 (76)		
	Total	100.0 (201)		
Collectively can influence area decisions	Agree	90.2 (193)	87.9 (189)	85.9 (177)
	Neither	8.4 (18)	10.2 (22)	11.2 (23)
	Disagree	1.4 (3)	1.9 (4)	2.9 (6)
	Total	100.0 (214)	100.0 (215)	100.0 (206)
Willing to help each other	Agree	88.4 (176)	78.7 (155)	89.4 (178)
	Disagree	11.6 (23)	21.3 (42)	10.6 (21)
	Total	100.0 (199)	100.0 (205)	100.0 (193)
Resident	No	20.3 (44)	21.7 (46)	23.3 (50)
	Yes	79.7 (173)	78.3 (166)	76.7 (165)
	Total	100.0 (217)	100.0 (212)	100.0 (182)
Hours volunteered/week	Mean ± SD (*n*)	7.2 ± 7.4 (183)	8.4 ± 9.4 (202)	7.7 ± 7.8 (182)
Mental wellbeing (SWEMWBS)	Mean ± SD	24. 5 ± 4.5 (199)	25.0 ± 4.0 (205)	23.9 ± 4.0 (193)

Total		100.0 (209)	100.0 (208)	100.0 (203)

Table 2 shows the final fully adjusted parsimonious model showing the associations between our explanatory factors and our primary outcome of mental wellbeing (SWEMWBS) amongst the BL partnership members. The results show that compared with baseline status in 2016, there was a significant 1.46 (0.14, 2.77) unit improvement in mental wellbeing at wave 2 in 2018 but there was no significant difference at wave 3 in 2020 (0.06 [−1.41, 1.53]). Across all waves, respondents who perceived that people in the area are willing to help each other had a significantly higher mental wellbeing score of around 1 unit (1.09 [0.19, 2.0]). Similarly, across all waves, those who agreed that collectively they can influence decisions in the area had a mental wellbeing score > 3 units higher (3.36 [1.51, 5.21]). Hours volunteered was also associated with a small positive improvement in mental wellbeing (0.08 [0.03, 0.12) across all waves. Across all waves, there was no significant association between mental wellbeing and resident status: both residents and non-residents reported similar impacts from participation in the BL.

**Table 2:** Analysis of change in mental wellbeing (SWEMWBS) between baseline and wave 2 (2018) and wave 3 (2020) and factors associated with SWEMWBS (*n* = 217)

Variables	Categories	Estimate	CI: Lower	CI: Upper	P value
Intercept		20.310	17.718	22.926	<.0001
Time	2020	0.062	−1.409	1.533	0.934
	2018	**1.456**	**0.139**	**2.769**	**0.030**
	2016	Ref			
Age groups	≤29 years	−2.447	−6.447	1.554	0.230
	30–44 years	−1.400	−2.840	0.039	0.057
	45–64 years	**−0.927**	**−1.816**	**−0.038**	**0.041**
	65 and above	Ref			
Gender	Female	−0.210	−1.122	0.703	0.639
	Male	Ref			
Ethnicity	Non-White	0.380	−1.236	1.997	0.632
	White	Ref			
Highest Education	No degree	0.213	−0.686	1.112	0.642
	One or more degrees	Ref			
Collectively can influence area decisions	Agree	**3.363**	**1.512**	**5.213**	**<0.001**
	Neither	1.397	−0.644	3.437	0.179
	Disagree	Ref			
Residents are willing to help	Agree	**1.091**	**0.187**	**1.996**	**0.018**
	Disagree	Ref			
Resident	Resident	0.831	−0.668	2.350	0.282
	Non-resident	Ref			
Time×Resident	2020 versus 2016 diff	−1.566	−3.224	0.091	0.060
	2018 versus 2016 diff	**−1.530**	**−2.991**	**−0.068**	**0.037**
	Baseline difference	Ref			
Hours volunteered/week		**0.075**	**0.033**	**0.118**	**0.001**

Statistically significant results with *p*-value <0.05, along with estimate and confidence intervals, are shown in bold.

The subgroup analysis by education—which used similar models as the main analysis—found that there was no significant difference in 2018 or 2020 in mental wellbeing for those without a degree-level education (Table 3). However, for those with one or more degrees, a proxy for higher socio-economic status (Table 4), there was nearly a 2-unit improvement (1.99 [0.14, 3.84]) in mental wellbeing in 2018, but no significant difference in 2020 (0.47 [−1.38, 2.32]). In both educational groups, those participants who agreed that they had collective control had a > 2-unit higher mental wellbeing score than those who did not (no degree = 2.77 [0.38, 5.17]), degree = 2.71 [0.02, 5.41]). Likewise, in both groups, for each hour spent volunteering in BL, there was a very small increase in mental wellbeing (with degree = 0.11 [0.04, 0.18]; no degree = 0.06 [0.01, 0.11]). Mental wellbeing scores for residents with a degree were higher (by 2.29 units [0.22, 4.36]) compared with non-residents with a degree.

**Table 3:** Analysis of change in mental wellbeing (SWEMWBS) between baseline and wave 2 (2018) and wave 3 (2020) and factors associated with SWEMWBS, for participants without a degree level education

Variables	Categories	Estimate	CI: Lower	CI: Upper	P value
Intercept		22.437	19.090	25.785	<0.001
Time	2020	−0.189	−2.458	2.080	0.870
	2018	1.018	−0.998	3.033	0.320
	2016	Ref			
Age groups	≤29	**−4.875**	**−9.071**	**−0.679**	**0.023**
	30–44 years	−1.766	−3.865	0.333	0.099
	45–64 years	**−1.461**	**−2.575**	**−0.346**	**0.010**
	≥65 years	Ref			
Ethnicity	Non-White	2.045	−0.333	4.423	0.091
	White	Ref			
Gender	Female	−0.034	−1.143	1.075	0.952
	Male	Ref			
Willing to help each other	Agree	0.968	−0.158	2.094	0.091
	Disagree	Ref			
Collectively can influence area decisions	Neither	0.410	−2.261	3.081	0.762
	Agree	**2.772**	**0.378**	**5.166**	**0.024**
	Disagree	Ref			
Resident	Resident	−0.185	−2.406	2.035	0.869
	Non-resident	Ref			
Time×Resident	2018 diff versus 2016 diff	−0.990	−3.241	1.262	0.387
	2020 diff versus 2016 diff	−1.654	−4.221	0.912	0.205
	2016 diff (Res versus Non-res)	Ref			
Hours volunteered/week		**0.063**	**0.011**	**0.114**	**0.017**

Statistically significant results with *p*-value <0.05, along with estimate and confidence intervals, are shown in bold.

**Table 4:** Analysis of change in mental wellbeing (SWEMWBS) between baseline and wave 2 (2018) and wave 3 (2020) and factors associated with SWEMWBS, for those with a degree level education

Variables	TIME	Estimate	Ci: Lower	CI: Upper	P value
Intercept		19.063	14.962	23.163	<0.001
Time	2020	0.471	−1.382	2.323	0.616
	2018	**1.990**	**0.141**	**3.840**	**0.035**
	2016	Ref			
	≤29	8.235	−0.409	16.879	0.062
Age groups	30–44 years	−1.012	−3.076	1.052	0.333
	45–64 years	−0.200	−1.648	1.247	0.784
	≥65 years	Ref			
	Non-White	−1.508	−3.716	0.701	0.179
Ethnicity	White	Ref			
	Female	−0.243	−1.728	1.242	0.746
Gender	Male	Ref			
	Agree	1.350	−0.142	2.842	0.076
Willing to help each other	Disagree	Ref			
	Neither	1.392	−1.436	4.220	0.332
Collectively can influence area decisions	Agree	**2.713**	**0.017**	**5.410**	**0.049**
	Disagree	Ref			
Resident	Resident	**2.289**	**0.215**	**4.363**	**0.031**
	Non-resident	Ref			
Time×Resident	2018 diff versus 2016 diff	**−2.289**	**−4.336**	**−0.241**	**0.029**
	2020 diff versus 2016 diff	−1.380	−3.455	0.695	0.190
	2016 diff (Res versus non-res)	Ref			
Hours volunteered/week		**0.110**	**0.036**	**0.183**	**0.004**

Statistically significant results with *p*-value <0.05, along with estimate and confidence intervals, are shown in bold.

The subgroup analysis by gender—which used similar models as the main analysis—found no significant difference in 2018 or 2020 in mental wellbeing for women (Supplementary Table A). Men had a significant increase of > 2 units (2.41 [0.55, 4.28]) in mental wellbeing score in 2018 but not 2020 (Supplementary Table B). The mental wellbeing score amongst men agreeing that they had collective control compared with men who did not was almost 5 units higher (4.91 [2.36, 7.46]). For women, mental wellbeing was significantly higher by 1.6 units (1.64 [0.48, 2.79]) amongst those who agreed that people in the area are willing to help each other. For men—but not women—there was also a small (0.20 [0.12, 0.28]) positive association between hours volunteered and mental wellbeing.

## Discussion

### Main findings of this study

In this small uncontrolled longitudinal study, we found that there was a statistically significant increase in mental wellbeing for all respondents in 2018 but that this declined again by 2020. This suggests that being involved in the BL potentially had a small positive impact on wellbeing in the medium term (2018) but not over the longer term (2020). However, this might be explained by the fact that the 2020 survey was delivered in summer 2020, during the COVID-19 pandemic when the mental wellbeing of the whole country had declined. Respondents who perceived that people in the area were willing to help each other had a significantly higher wellbeing score as did those who agreed that collectively they could influence decisions in the area. Hours volunteered also had a small positive association with increased mental wellbeing for men. This suggests that increased involvement in the BL intervention both in terms of time and influence was associated with increases in mental wellbeing. This may have been a result of the direct (for example influencing what happens in their neighbourhood) or indirect (e.g. higher social cohesion) pathways potentially influenced by the BL. In terms of our sub-group analysis, among those with ‘no degree’, there was no significant change in mental wellbeing score in 2018 or 2020, but there was a significant improvement in 2018 among those who had one or more degrees. However, for both educational groups, those who felt they could collectively influence area decisions, had a positive increase in mental wellbeing. For both educational groups, there was also a small significant positive association between hours volunteered and mental wellbeing amongst men. In terms of gender, there was no significant difference in mental wellbeing for women, whereas men had a significant increase in 2018.

### What is already known on this topic

Previous research has found, like ours, that collective control and social cohesion are important mechanisms through which area-based interventions impact on mental wellbeing. For example, a survey of communities undergoing regeneration in Glasgow found that residents’ perceptions of their ability to influence decisions about where they lived were positively associated with mental health outcomes.^14^ Similarly, a study of neighbourhood belonging found moderate associations with wellbeing stemming from greater social participation and increased feelings of belonging to the neighbourhood.^15^ Our results also suggest that increased involvement in the BL intervention increased mental wellbeing. This is in keeping with other studies of volunteering which have found a relationship between the amount of time spent volunteering and health benefits.^16^ We also found evidence of unequal impacts: only higher educated participants experienced an increase in mental wellbeing and men’s wellbeing increased, whereas women’s did not. This is in keeping with previous research which found that there may be negative or unintended consequences if initiatives succeed in building social trust within communities and engaging people in collective action, but gains are not equally distributed—with residents from higher socio-economic backgrounds having greater benefits.^17^ Indeed, some studies have reported that living in areas with higher elements of social cohesion can actually be harmful for excluded residents.^18^ More generally, our findings are also consistent with theory that suggests factors such as control and feeling part of a neighbourhood can help to facilitate collective action in pursuit of shared goals.^19^

### What this study adds

This research provides a longitudinal analysis of the role that community empowerment interventions can have on health. Our findings, showing an association between collective control, social cohesion and mental wellbeing, suggest that although participation and volunteering matters for health so too does feeling that it is possible to have influence over local decisions. This adds to the literature which suggests that increasing collective control over decisions affecting where communities live could be a potential pathway to better health. However, our results also add a note of caution that not all participants may benefit equally from involvement.^17^^,^^18^ This has important implications for the future intervention design and implementation.

### Limitations of this study

This study is subject to a number of important limitations. Firstly, the sample is small and restricts statistical power. Secondly, the survey population is skewed towards the highly educated, White people and older age groups. This is unsurprising given the evidence that shows how people from more disadvantaged social backgrounds or those with poor health are less likely to participate in volunteering activities.^20^ But, it does further limit generalizability. Thirdly, the study relies on self-reported outcomes which may limit the precision and reliability of our findings. However, there is evidence that shows a strong association between self-reported health and more objective outcomes such as mortality.^21^ Fourthly, our nested cohort analysis assumed that data were missing at random which might not be the case (although in our examination only sex predicted missingness so we adjusted for this in the main models). Fifthly, the study had no control group and this restricts our ability to conclude that the changes observed were related to participation in BL. Our study can therefore only assess correlation between participation, collective control, etc. and mental wellbeing—it cannot establish causation. Finally, the impact of the COVID-19 pandemic is an extremely important—and unforeseeable—issue in our trend data as the bias it creates potentially affects both the outcome and all of the explanatory variables too (control, willingness, hours of volunteering). It is therefore very difficult to interpret the 2020 survey data. Indeed, other research has suggested that the pandemic and the associated restrictions negatively impacted on mental wellbeing.^22^

## Conclusion

This research significantly extends the empirical evidence base on the health effects of community empowerment interventions by determining—using a longitudinal design—that collective control and social cohesion are associated with better mental wellbeing amongst people actively involved in the BL in relatively disadvantaged areas. It has also found that the health benefits of involvement in BL were greater amongst men and those from higher educational backgrounds. This suggests that additional care must be taken in future interventions to ensure that benefits are equally distributed. Future design, implementation and evaluation work should explore these issues further.

## Contributors

N.A. led the data analysis, interpretation and led the writing up of findings with C.B. V.J.M. led write up of background literature with C.B., contributed to interpretation of findings and prepared manuscript for publication. E.H. contributed to the interpretation of the findings and background literature. J.P. conceived the idea for the overall CiC study, led on the planning and development of the study and advised on data collection, analysis and interpretation. C.B. and A.K. conceived design of the research reported here and advised on analysis and interpretation. All authors contributed to the writing of the article and approved the final version.

## Supplementary Material

JPH_Tables_fdac073

## Notes

**N Akhter**,

**VJ McGowan**,

**E Halliday**,

**J Popay**,

**A Kasim**,

**C Bambra**,

## References

[1] South J, Stansfield J, Mehta P. A guide to community-centred approaches for health and wellbeing, Public Health England: London. 2015.

[2] Syme SL . Control and health: a personal perspective. In: Steptoe A, Appels A (eds), Stress, Personal Control and Health. Oxford, England: John Wiley & Sons, 1989, 3–18.

[3] Siegrist J, Marmot M. Health inequalities and the psychosocial environment - two scientific challenges. Soc Sci Med 2004;58:1463–73. 14759690 10.1016/S0277-9536(03)00349-6

[4] Woodall J, Raine G, South J, and Warwick-Booth L, (2010) Empowerment & health and well-being: evidence review. Project Report. Centre for Health Promotion Research, Leeds Metropolitan University.

[5] Popay J, Whitehead M, Ponsford R et al. Power, control, communities and health inequalities I: theories, concepts and analytical frameworks. Health Promot Int 2020;36:1253–1263. 10.1093/heapro/daaa133 PMC8515177 33382890

[6] Marmot M . Status Syndrome: How Your Place on the Social Gradient Directly Affects Your Health. London: Bloomsbury Paperbacks, 2015.

[7] Orton L, Pennington A, Nayak S et al. Group-based microfinance for collective empowerment: a systematic review of health impacts. Bull World Health Organ 2016;94(9):694–704A. 27708475 10.2471/BLT.15.168252 PMC5034638

[8] Milton B, Attree P, French B et al. The impact of community engagement on health and social outcomes: a systematic review. Community Dev J 2012. 10.1093/cdj/bsr043.

[9] Diderichsen F, Evans T, Whitehead M. The social basis of disparities in health. In: Evans T et al., eds. *Challenging Inequities in Health*. New York, Oxford UP, 2001. 10.1093/acprof:oso/9780195137408.003.0002.

[10] Whitehead M, Pennington A, Orton L et al. How could differences in “control over destiny” lead to socio-economic inequalities in health? A synthesis of theories and pathways in the living environment. Health Place 2016;39:51–61. 10.1016/j.healthplace.2016.02.002 26986982

[11] Egan M, Abba K, Barnes A et al. Building collective control and improving health through a place-based community empowerment initiative: qualitative evidence from communities seeking agency over their built environment. Crit Public Health 2021;31:268–279.

[12] McGowan VJ, Akhter N, Halliday E et al. Collective control, social cohesion and health and well-being: baseline survey results from the communities in control study in England. J Public Health (Oxf) 2021. 10.1093/pubmed/fdaa227. 33423066

[13] McGowan VJ, Wistow J, Lewis SJ et al. Pathways to mental health improvement in a community-led area-based empowerment initiative: evidence from the big local “communities in control” study, England. J Public Health (Oxf) 2019;41(4):850–857. 10.1093/pubmed/fdy192 31034020

[14] Baba C, Kearns A, McIntosh E et al. Is empowerment a route to improving mental health and wellbeing in an urban regeneration (UR) context? Urban Stud 2017;54:1619–1637.

[15] Elliott J, Gale CR, Parsons S, Kuh D. Neighbourhood cohesion and mental wellbeing among older adults: a mixed methods approach. Soc Sci Med 2014;107:44–51. 24602970 10.1016/j.socscimed.2014.02.027

[16] Jenkinson CE, Dickens AP, Jones K et al. Is volunteering a public health intervention? A systematic review and meta-analysis of the health and survival of volunteers. BMC Public Health 2013;13(1):773. 23968220 10.1186/1471-2458-13-773 PMC3766013

[17] Portes A . Social capital: its origins and applications in modern sociology. Annu Rev Sociol 1998;24(1):1–24.

[18] Villalonga-Olives E, Kawachi I. The dark side of social capital: a systematic review of the negative health effects of social capital. Soc Sci Med 2017;194:105–27. 29100136 10.1016/j.socscimed.2017.10.020

[19] Chinn D . Critical health literacy: a review and critical analysis. Soc Sci Med 2011;73:60–7. 10.1016/j.socscimed.2011.04.004 21640456

[20] McMunn A, Nazroo J, Wahrendorf M et al. Participation in socially-productive activities, reciprocity and wellbeing in later life: baseline results in England. Ageing Soc 2009;29:765–782.

[21] Norman PD, Bambra C. Incapacity or unemployment? The utility of an administrative data source as an updatable indicator of population health. Popul Space Place 2007;13:333–352.

[22] Bambra C, Munford L, Alexandros A, et al. COVID-19 and the Northern Powerhouse. Northern Health Science Alliance, Newcastle.; 2020. https://www.thenhsa.co.uk/app/uploads/2020/11/NP-COVID-REPORT-101120-.pdf.

