# Supplementary material for: Community empowerment and mental wellbeing: longitudinal findings from a survey of people actively involved in the big local place-based initiative in England
Source: J Public Health (Oxf). 2022 Jul 30;45(2):423–31. doi: 10.1093/pubmed/fdac073 (PMC10273354; doi:10.1093/pubmed/fdac073)
Supplement: JPH_Tables_fdac073 [file jph_tables_fdac073.docx]

## Table 1: Socio-Demographic, Health and Explanatory data for cohort participants (n= 217)

| **Variable** | **Categories** | **2016**  **% (n)** | **2018**  **% (n)** | **2020**  **% (n)** |
| --- | --- | --- | --- | --- |
| Age | <=29 years | 0.5 (1) |  |  |
|  | 30-44 years | 15.7 (34) |  |  |
|  | 45-64 years | 50.5 (109) |  |  |
|  | >=65 years | 33.3 (72) |  |  |
|  | Total | 100.0 (216) |  |  |
| Sex | Female | 56.0 (121) |  |  |
|  | Male | 44.0 (95) |  |  |
|  | Total | 100.0 (216) |  |  |
| Ethnicity | BAME | 8.5 (18) |  |  |
|  | White | 91.5 (194) |  |  |
|  | Total | 100.0 (212) |  |  |
| Highest education | No degree | 62.2 (125) |  |  |
|  | Degree | 37.8 (76) |  |  |
|  | Total | 100.0 (201) |  |  |

| Collectively can influence area decisions | Agree | 90.2 (193) | 87.9 (189) | 85.9 (177) |
| --- | --- | --- | --- | --- |
|  | Neither | 8.4 (18) | 10.2 (22) | 11.2 (23) |
|  | Disagree | 1.4 (3) | 1.9 (4) | 2.9 (6) |
|  | Total | 100.0 (214) | 100.0 (215) | 100.0 (206) |
| Willing to help each other | Agree | 88.4 (176) | 78.7 (155) | 89.4 (178) |
|  | Disagree | 11.6 (23) | 21.3 (42) | 10.6 (21) |
|  | Total | 100.0 (199) | 100.0 (205) | 100.0 (193) |
| Resident | No | 20.3 (44) | 21.7 (46) | 23.3 (50) |
|  | Yes | 79.7 (173) | 78.3 (166) | 76.7 (165) |
|  | Total | 100.0 (217) | 100.0 (212) | 100.0 (182) |
| Hours volunteered/ week | Mean ± SD (n) | 7.2 ± 7.4 (183) | 8.4 ± 9.4 (202) | 7.7 ± 7.8 (182) |
| Mental wellbeing (SWEMWBS) | Mean ± SD | 24. 5 ± 4.5 (199) | 25.0 ± 4.0 (205) | 23.9 ± 4.0 (193) |

| Total |  | 100.0 (209) | 100.0 (208) | 100.0 (203) |
| --- | --- | --- | --- | --- |

## Table 2: Analysis of change in mental wellbeing (SWEMWBS) between baseline and wave 2 (2018) and wave 3 (2020) and factors associated with SWEMWBS (n=217)

| **Variables** | **Categories** | **Estimate** | **CI: Lower** | **CI: Upper** | **P value** |
| --- | --- | --- | --- | --- | --- |
| Intercept |  | 20.310 | 17.718 | 22.926 | <.0001 |
| Time | 2020 | 0.062 | -1.409 | 1.533 | 0.934 |
|  | 2018 | **1.456** | **0.139** | **2.769** | **0.030** |
|  | 2016 | Ref |  |  |  |
| Age groups | <=29 years | -2.447 | -6.447 | 1.554 | 0.230 |
|  | 30-44 years | -1.400 | -2.840 | 0.039 | 0.057 |
|  | 45-64 years | **-0.927** | **-1.816** | **-0.038** | **0.041** |
|  | 65 and above | Ref |  |  |  |
| Gender | Female | -0.210 | -1.122 | 0.703 | 0.639 |
|  | Male | Ref |  |  |  |
| Ethnicity | Non-White | 0.380 | -1.236 | 1.997 | 0.632 |
|  | White | Ref |  |  |  |
| Highest Education | No degree | 0.213 | -0.686 | 1.112 | 0.642 |
|  | One or more degrees | Ref |  |  |  |
| Collectively can influence area decisions | Agree | **3.363** | **1.512** | **5.213** | **<0.001** |
|  | Neither | 1.397 | -0.644 | 3.437 | 0.179 |
|  | Disagree | Ref |  |  |  |
| Residents are willing to help | Agree | **1.091** | **0.187** | **1.996** | **0.018** |
|  | Disagree | Ref |  |  |  |
| Resident | Resident | 0.831 | -0.668 | 2.350 | 0.282 |
|  | Non-resident | Ref |  |  |  |
| Time*Resident | 2020 vs 2016 diff | -1.566 | -3.224 | 0.091 | 0.060 |
|  | 2018 vs 2016 diff | **-1.530** | **-2.991** | **-0.068** | **0.037** |
|  | Baseline difference | Ref |  |  |  |
| Hours volunteered/ week |  | **0.075** | **0.033** | **0.118** | **0.001** |

## Table 3: Analysis of change in mental wellbeing (SWEMWBS) between baseline and wave 2 (2018) and wave 3 (2020) and factors associated with SWEMWBS, for participants without a degree level education

| **Variables** | **Categories** | **Estimate** | **CI: Lower** | **CI: Upper** | **P-value** |
| --- | --- | --- | --- | --- | --- |
| Intercept |  | 22.437 | 19.090 | 25.785 | <0.001 |
| Time | 2020 | -0.189 | -2.458 | 2.080 | 0.870 |
|  | 2018 | 1.018 | -0.998 | 3.033 | 0.320 |
|  | 2016 | Ref |  |  |  |
| Age groups | <=29 | **-4.875** | **-9.071** | **-0.679** | **0.023** |
|  | 30-44 years | -1.766 | -3.865 | 0.333 | 0.099 |
|  | 45-64 years | **-1.461** | **-2.575** | **-0.346** | **0.010** |
|  | >=65 years | Ref |  |  |  |
| Ethnicity | Non-White | 2.045 | -0.333 | 4.423 | 0.091 |
|  | White | Ref |  |  |  |
| Gender | Female | -0.034 | -1.143 | 1.075 | 0.952 |
|  | Male | Ref |  |  |  |
| Willing to help each other | Agree | 0.968 | -0.158 | 2.094 | 0.091 |
|  | Disagree | Ref |  |  |  |
| Collectively can influence area decisions | Neither | 0.410 | -2.261 | 3.081 | 0.762 |
|  | Agree | **2.772** | **0.378** | **5.166** | **0.024** |
|  | Disagree | Ref |  |  |  |
| Resident | Resident | -0.185 | -2.406 | 2.035 | 0.869 |
|  | Non-resident | Ref |  |  |  |
| Time*Resident | 2018 diff vs 2016 diff | -0.990 | -3.241 | 1.262 | 0.387 |
|  | 2020 diff vs 2016 diff | -1.654 | -4.221 | 0.912 | 0.205 |
|  | 2016 diff (Res vs Non-res) | Ref |  |  |  |
| Hours volunteered/ week |  | **0.063** | **0.011** | **0.114** | **0.017** |

## Table 4: Analysis of change in mental wellbeing (SWEMWBS) between baseline and wave 2 (2018) and wave 3 (2020) and factors associated with SWEMWBS, for those with a degree level education

| **Variables** | **TIME** | **Estimate** | **Ci: Lower** | **CI: Upper** | **P-value** |
| --- | --- | --- | --- | --- | --- |
| Intercept |  | 19.063 | 14.962 | 23.163 | <0.001 |
| Time | 2020 | 0.471 | -1.382 | 2.323 | 0.616 |
|  | 2018 | **1.990** | **0.141** | **3.840** | **0.035** |
|  | 2016 | Ref |  |  |  |
|  | <=29 | 8.235 | -0.409 | 16.879 | 0.062 |
| Age groups | 30-44 years | -1.012 | -3.076 | 1.052 | 0.333 |
|  | 45-64 years | -0.200 | -1.648 | 1.247 | 0.784 |
|  | >=65 years | Ref |  |  |  |
|  | Non-White | -1.508 | -3.716 | 0.701 | 0.179 |
| Ethnicity | White | Ref |  |  |  |
|  | Female | -0.243 | -1.728 | 1.242 | 0.746 |
| Gender | Male | Ref |  |  |  |
|  | Agree | 1.350 | -0.142 | 2.842 | 0.076 |
| Willing to help each other | Disagree | Ref |  |  |  |
|  | Neither | 1.392 | -1.436 | 4.220 | 0.332 |
| Collectively can influence area decisions | Agree | **2.713** | **0.017** | **5.410** | **0.049** |
|  | Disagree | Ref |  |  |  |
| Resident | Resident | **2.289** | **0.215** | **4.363** | **0.031** |
|  | Non-resident | Ref |  |  |  |
| Time*Resident | 2018 diff vs 2016 diff | **-2.289** | **-4.336** | **-0.241** | **0.029** |
|  | 2020 diff vs 2016 diff | -1.380 | -3.455 | 0.695 | 0.190 |
|  | 2016 diff (Res vs non-res) | Ref |  |  |  |
| Hours volunteered/ week |  | **0.110** | **0.036** | **0.183** | **0.004** |

## Supplementary Table A: Analysis of change in mental wellbeing (SWEMWBS) between baseline and wave 2 (2018) and wave 3 (2020) and factors associated with SWEMWBS, women

| **Variables** | **Categories** | **Estimate** | **CI: Lower** | **CI: Upper** | **P value** |
| --- | --- | --- | --- | --- | --- |
| Intercept |  | 22.228 | 18.666 | 25.790 | <0.001 |
| Time | 2018 | 0.086 | -1.802 | 1.974 | 0.929 |
|  | 2020 | -0.659 | -2.735 | 1.416 | 0.531 |
|  | 2016 | Ref |  |  |  |
| Age groups | <=29 years | NA |  |  |  |
|  | 30-44 years | **-1.904** | **-3.722** | **-0.086** | **0.040** |
|  | 45-64 years | **-1.567** | **-2.721** | **-0.413** | **0.008** |
|  | >=65 years | Ref |  |  |  |
| Ethnicity | Non-White | -0.480 | -2.573 | 1.613 | 0.651 |
|  | White | Ref |  |  |  |
| Highest Education | No degree | 0.508 | -0.633 | 1.649 | 0.381 |
|  | One or more degrees | Ref |  |  |  |
| Willing to help each other | Agree | **1.635** | **0.480** | **2.790** | **0.006** |
|  | Disagree | Ref |  |  |  |
| Collectively can influence area decisions | Agree | 1.403 | -1.321 | 4.127 | 0.311 |
|  | Neither | 0.524 | -2.419 | 3.468 | 0.725 |
|  | Disagree | Ref |  |  |  |
| Resident | Resident | 1.003 | -1.074 | 3.079 | 0.342 |
|  | Non-resident | Ref |  |  |  |
| Time*Resident | 208 diff vs 2016 diff | -0.295 | -2.375 | 1.785 | 0.780 |
|  | 2020 diff vs 2016 diff | -1.048 | -3.397 | 1.301 | 0.380 |
|  | 206 diff (Res vs non-res) | Ref |  |  |  |
| Hours volunteered/ week | | 0.024 | -0.023 | 0.072 | 0.308 |

## Supplementary Table B: Analysis of change in mental wellbeing (SWEMWBS) between baseline and wave 2 (2018) and wave 3 (2020) and factors associated with SWEMWBS, men

| **Variables** | **Categories** | **Estimate** | **CI-Lower** | **CI-Upper** | **P-value** |
| --- | --- | --- | --- | --- | --- |
| Intercept |  | 17.371 | 13.541 | 21.200 | <0.001 |
| Time | 2020 | 0.847 | -1.256 | 2.950 | 0.427 |
|  | 2018 | **2.412** | **0.547** | **4.277** | **0.012** |
|  | 2016 | Ref |  |  |  |
| Age groups | <=29 | -2.712 | -6.555 | 1.130 | 0.165 |
|  | 30-44 years | -0.863 | -3.119 | 1.393 | 0.450 |
|  | 45-64 years | -0.222 | -1.459 | 1.016 | 0.723 |
|  | >=65 years | Ref |  |  |  |
| Ethnicity | Non-White | 0.792 | -1.488 | 3.073 | 0.493 |
|  | White | Ref |  |  |  |
| Highest Education | No degree | -0.033 | -1.301 | 1.234 | 0.959 |
|  | One or more degrees | Ref |  |  |  |
| Willing to help each other | Agree | 1.326 | -0.057 | 2.708 | 0.060 |
|  | Disagree | Ref |  |  |  |
| Collectively can influence area decisions | Agree | **4.909** | **2.356** | **7.463** | **<0.001** |
|  | Neither | 2.048 | -0.812 | 4.907 | 0.159 |
|  | Disagree | Ref |  |  |  |
| Resident | Resident | 0.849 | -1.329 | 3.027 | 0.442 |
|  | Non-resident | Ref |  |  |  |
| Time*Resident | 2018 diff vs 2016 diff | **-2.214** | **-4.319** | **-0.110** | **0.039** |
|  | 2020 diff vs 2016 diff | -2.263 | -4.616 | 0.090 | 0.059 |
|  | 2016 diff (Res vs non-res) | Ref |  |  |  |
| Hours volunteered/ week |  | **0.198** | **0.119** | **0.276** | **<0.001** |
